# Telangiectasia Macularis Eruptiva Perstans Following Radiotherapy: Atypical Presentation of a Rare Condition

**DOI:** 10.7759/cureus.84675

**Published:** 2025-05-23

**Authors:** Mariam Harb, Anne Theunis, Evelyne Berlingin

**Affiliations:** 1 Dermatology, Chu Helora Hospital, Mons, BEL; 2 Pathology and Laboratory Medicine, Erasmus Hospital, Brussels, BEL

**Keywords:** clinical dermatology, clinical pathology, mast cell disorder, primary breast malignancy, radiotherapy (rt)

## Abstract

Telangiectasia macularis eruptiva perstans (TMEP) is a rare form of cutaneous mastocytosis characterized by telangiectatic macules and plaques. We report an atypical case of TMEP in an 80-year-old female with a history of left invasive lobular carcinoma, previously treated with mastectomy, radiotherapy, and hormonal therapy. The patient presented with a single telangiectatic plaque on the left side of her trunk. Histopathological examination confirmed the diagnosis of TMEP.

This case is notable due to the patient's advanced age and the history of extensive cancer treatment, including radiation therapy. The potential association between radiation therapy and the development of mast cell disorders is explored, considering the possible role of radiation-induced skin changes in the pathogenesis of TMEP. This case underscores the importance of considering TMEP in the differential diagnosis of telangiectatic lesions, particularly in patients with a history of malignancy and radiation therapy. Further research is warranted to elucidate the mechanisms linking radiation therapy to mast cell proliferation and activation.

## Introduction

Telangiectasia macularis eruptiva perstans (TMEP) is a rare variant of cutaneous mastocytosis characterized by telangiectatic macules and plaques, typically occurring in adults and associated with increased dermal mast cell proliferation [1]. While TMEP is traditionally regarded as a cutaneous-limited form, systemic involvement can occur in a subset of cases, highlighting the importance of accurate diagnosis and clinical monitoring [1,2].

This case report presents an atypical manifestation of TMEP in a patient with a history of breast cancer treated with hormonal therapy and radiotherapy. The association between radiation therapy and mast cell-related disorders has been documented in a limited number of reports, suggesting that ionizing radiation may serve as a local trigger for mast cell accumulation or activation [1-3]. Proposed mechanisms include the release of inflammatory mediators in response to radiation-induced tissue damage, disruption of skin barrier integrity, and the Koebner phenomenon [3-6]. Although rare, several published cases have reported the development of cutaneous mastocytosis in patients with breast carcinoma following radiotherapy, with most lesions occurring within or near the irradiated field [1,2,5-7].

## Case presentation

An 80-year-old female with a history of left invasive lobular carcinoma underwent a total mastectomy in September 2022, followed by five sessions of radiotherapy completed in November 2022 and adjuvant hormonal therapy. Shortly after, she experienced a burning sensation extending from the left mid-axillary line to the mid-clavicular line.

**Figure 1:**
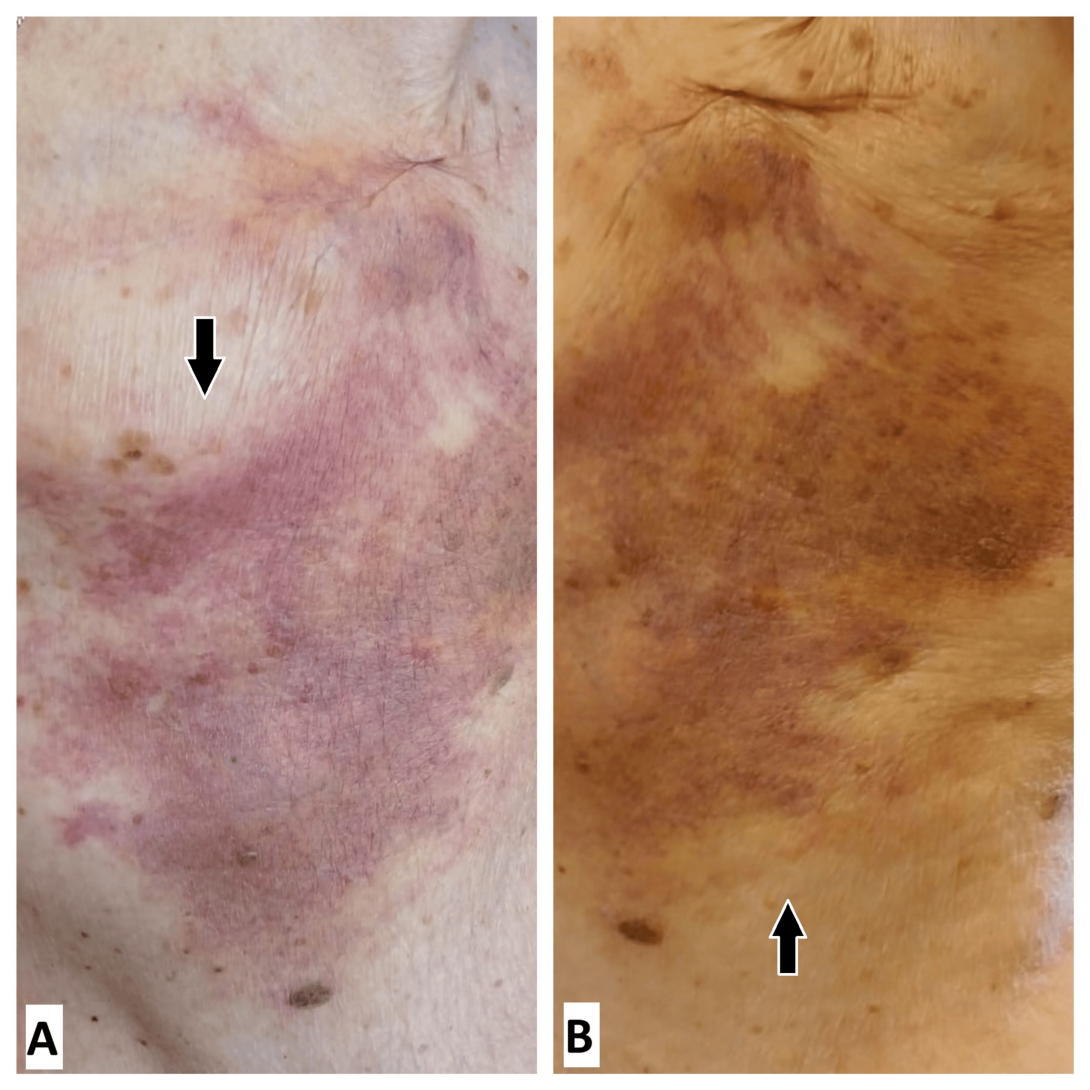
A and B: Poorly defined erythematous and violaceous plaque with a telangiectatic surface

In October 2024, she had surgery for a suspected left axillary lymph node, which revealed no metastasis. Two months later, she observed a poorly defined erythematous and violaceous plaque resembling an ecchymosis at the site of the burning sensation. This plaque had a telangiectatic surface and no history of trauma or associated symptoms indicative of histamine release (Figures 1A, 1B). Our differential diagnosis was angiosarcoma, breast cancer relapse, or eczema. 

**Figure 2:**
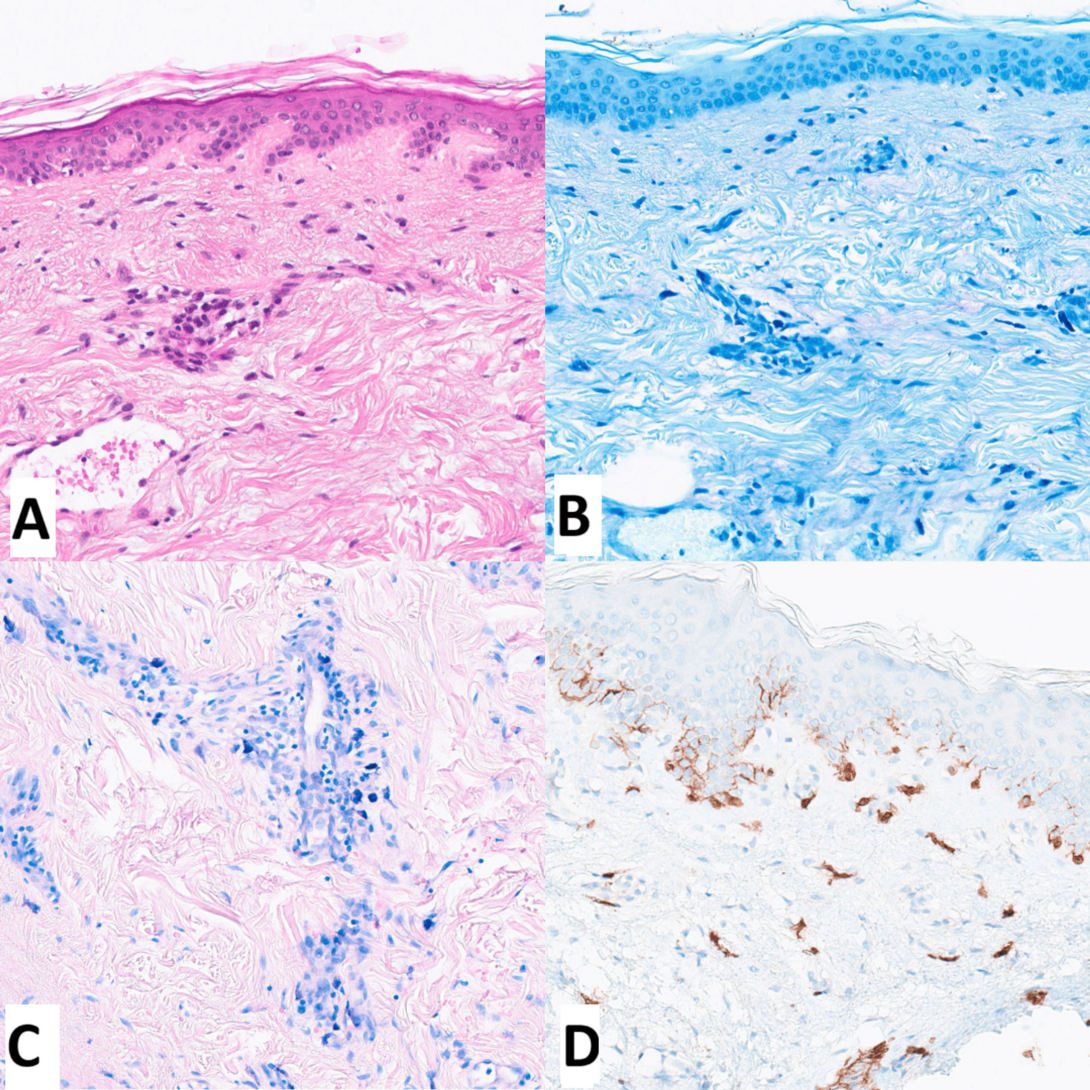
A, B, and C: Hematoxylin-eosin, bleu de toluidine, and Giemsa colorations showing an edematous dermis, ectatic capillaries, and a dense mast cell infiltrate, and 2D immunohistochemistry showing CD117 positivity.

Diagnostic investigations for the plague included several tests. Blood work showed moderate thrombocytopenia (163,000/microL; normal level is more than 175,000/microL), slightly elevated tryptase levels (8.8 microg/L; normal is under 8 microg/L), and high total IgE (500 kU/L; normal level is less than 150 kU/L). A cutaneous biopsy demonstrated an edematous dermis, ectatic capillaries, and a dense mast cell infiltrate of over 30 cells per high-power field. Immunohistochemical analysis confirmed CD117 positivity and a mild eosinophilic infiltrate, leading to a diagnosis of telangiectatic mastocytosis (Figures 2A-2D). Genetic testing for the D816V mutation in the KIT gene was negative. A positron emission tomography (PET) scan was conducted to rule out metastasis, and it showed no suspicious lesions. The patient received antihistamine treatment, resulting in the resolution of the lesion within one month. 

## Discussion

Telangiectasia macularis eruptiva perstans (TMEP) is a rare variant of cutaneous mastocytosis (CM). While it has been regarded as limited to the skin, recent studies indicate that systemic involvement may be present in up to 47% of cases, with aggressive systemic mastocytosis occurring in approximately 9% [1,2].

Mastocytosis is a heterogeneous group of disorders characterized by the accumulation of clonal mast cells in various tissues, including the skin and bone marrow. The clinical manifestations are due to the release of mast cell mediators and, less frequently, due to the destructive infiltration of mast cells. The diagnosis of mastocytosis is based on the World Health Organization criteria, which include elevated serum tryptase levels, histopathological and immunophenotypic evaluation of mast cells, and molecular analysis for KIT mutations, particularly the D816V mutation [3].

Multiple studies have reported cutaneous mastocytosis in association with radiation therapy in breast cancer patients. For example, Murphy et al. described a case of post-radiotherapy cutaneous mastocytosis in a patient with breast carcinoma, suggesting that mast cell accumulation may occur secondary to local mediators produced in response to radiation damage. This phenomenon, although rare, underscores the need for clinicians to consider mastocytosis in patients presenting with rashes post-radiotherapy. There are seven previous reports in the literature discussing this association [1,4].

The patient's presentation of a burning sensation and erythematous rash post-mastectomy and radiotherapy raises the possibility of radiation-associated cutaneous mastocytosis. Given the potential for systemic involvement, further evaluation, including a bone marrow biopsy, may be warranted to rule out systemic mastocytosis [2].

Furthermore, the potential association between mastocytosis and cancer has been explored in the literature. While mastocytosis itself is not considered a malignancy, it can coexist with other hematologic and non-hematologic malignancies. For instance, systemic mastocytosis has been reported to occur concurrently with myeloid sarcoma, highlighting the importance of considering the possibility of multiple tumors in patients with mastocytosis. Additionally, there have been reports of TMEP associated with malignant melanoma and multiple myeloma, suggesting a possible link between mastocytosis and various malignancies [5-7].

## Conclusions

This case contributes to the growing recognition that radiotherapy may act as a potential trigger for localized mast cell proliferation, highlighting the need to consider cutaneous mastocytosis in the differential diagnosis of vascular skin lesions in cancer survivors. It underscores the importance of biopsy and immunohistochemistry in accurate diagnosis and suggests that external factors like radiation may play a role in the pathogenesis of certain forms of cutaneous mastocytosis, warranting further research into the mechanisms underlying this association.
